# Clinical Profile and Outcomes of Pediatric Snakebite Envenomation: A Three-Year Retrospective Study From a Rural Tertiary Care Center in South India

**DOI:** 10.7759/cureus.73976

**Published:** 2024-11-19

**Authors:** Tamilarasan Muniyapillai, Maniprabhu S, Abinaya R, Karthikeyan Kulothungan, Sriranganathan Thirunavukkarasu, Neethu George

**Affiliations:** 1 Community Medicine, Dhanalakshmi Srinivasan Medical College and Hospital, Perambalur, IND; 2 Community Medicine, K.A.P. Viswanatham Medical College, Trichy, IND; 3 Community Medicine, Panimalar Medical College Hospital and Research Institute, Chennai, IND; 4 Community Medicine, Srinivasan Medical College and Hospital, Trichy, IND

**Keywords:** anti-snake venom, neuroparalytic, outcome assessment, snakebite, snakebite envenoming, vasculotoxic

## Abstract

Background

Snakebite envenomation remains a significant public health challenge in tropical countries, particularly affecting the pediatric population. Children are especially vulnerable because of their smaller body mass, outdoor activities, and delayed presentation to healthcare facilities. This study aimed to analyze the clinical profile, demographic patterns, and envenomation characteristics of snakebites in children aged 1-16 years presenting to a tertiary care center. Additionally, the study sought to evaluate the spectrum of complications and clinical outcomes in pediatric snakebite cases while assessing the mortality rate and associated risk factors in pediatric snakebite envenomation.

Methodology

A retrospective medical record review was conducted analyzing all snakebite cases in children aged below 16 years admitted to the Department of Pediatrics, Dhanalakshmi Srinivasan Medical College and Hospital, Perambalur, Tamil Nadu (southern state in India), between January 2021 and December 2023. Data extracted included demographics, bite characteristics, clinical manifestations, time to healthcare presentation, and treatment details. Management protocols followed World Health Organization (WHO) guidelines for snakebite envenomation, including the administration of polyvalent anti-snake venom (ASV) when indicated.

Results

Among 202 pediatric snakebite cases, children aged 9-12 years constituted the majority (n = 110, 54.5%), with significant male predominance (n = 148, 73.3%). Unidentified snakes were responsible for the highest proportion of bites (n = 72, 35.6%), followed by vipers (n = 65, 32.2%) and cobras (n = 53, 26.2%). Lower limb bites were most frequent (n = 108, 53.5%), and seasonal analysis revealed peak incidence during January-April (n = 106, 52.5%). Common clinical manifestations included hematuria (n = 112, 55.4%), oliguria (n = 102, 50.5%), and renal failure (n = 80, 39.6%). Most patients (n = 120, 59.4%) received antivenom within six hours, with 46.6% (n = 94) requiring 5-10 vials. The overall mortality rate was 9.9% (n = 20). Statistical analysis revealed significant associations between mortality and snake species identification (p = 0.0014), with the highest mortality in unidentified snakebites (n = 15, 20.8%). Anatomical bite site (p = 0.042), renal failure (p = 0.001), respiratory paralysis (p = 0.001), and ptosis (p = 0.001) were also significantly associated with mortality. Time to antivenom administration significantly impacted survival (p = 0.001), with mortality rates of 0.8% (n = 1) for treatment within six hours, increasing to 38.5% (n = 10) for delays beyond 12 hours. Demographics and local manifestations showed no significant correlation with mortality.

Conclusion

Our study reveals distinct patterns in pediatric snakebites, predominantly affecting male children in pre-adolescent age. The high proportion of unidentified snakes remains challenging. With renal and hematological manifestations being frequent, mortality is significantly influenced by delayed treatment, respiratory paralysis, and renal failure. Early antivenom administration, proper snake identification, and prompt medical intervention remain crucial factors in improving outcomes and reducing mortality.

## Introduction

The World Health Organization (WHO) officially designated snakebite envenoming as a neglected tropical disease in 2017, and it remains a significant global health concern [1]. The annual incidence of snakebite affects a staggering five million individuals, primarily affecting rural populations living in the tropics and sub-tropics [2]. It is a significant contributor to both mortality and long-term disabilities, particularly among children residing in rural, underdeveloped regions [1]. Every year, worldwide statistics reveal that around four lakh individuals with disabilities are affected, resulting in an annual death toll of over 138,000 [3]. The seriousness of the matter lies in the intricate composition of snake venoms, which comprise over 100 different proteins and peptides. These substances, both toxic and non-toxic, exhibit specificity toward a diverse array of tissue receptors, resulting in clinically challenging situations with broad features and complications [4]. Neurotoxicity and coagulotoxicity are the predominant factors contributing to death in snakebite cases, emphasizing the importance of comprehending these snakebite syndromes [5].

People have known India as the "Land of Snake Charmers" for centuries [6]. Every year, snakebite deaths account for between 35 thousand and 50 thousand fatalities in India [7]. The most common age group for snakebites is school-age children, adolescents, and young adults. It is responsible for 3% of all pediatric deaths between the ages of five and 14 [6].

High rates of morbidity and mortality affect the communities in these areas because of restricted access to healthcare and a scarcity of anti-snake venom (ASV), the only specific treatment accessible. Tourniquets, suctioning the location of the bite, amputating the bitten finger, and applying herbal leaf extracts are still prevalent conventional remedies for snakebites in many developing countries. These techniques might serve as substantial indicators of mortality and morbidity [8]. Common vectors of snakebites include individuals engaged in agricultural labor, forest exploration, barefoot labor in fields, nocturnal labor, or traversing dark highways. Many of these vulnerable individuals reside in rural locations where access to healthcare is limited and live below the poverty line [9]. Snakebite is now a notifiable disease in the state of Tamil Nadu in India.

Children exhibit distinct vulnerabilities to snakebite envenomation compared to adults through multiple mechanisms. Their inherent curiosity combined with limited situational awareness and behavioral control increases their exposure risk to snakebite incidents [10]. Beyond anatomical differences such as body mass, developmental biochemical variations significantly influence snakebite outcomes. A critical factor in this context is developmental hemostasis, a physiological phenomenon characterized by age-dependent variations in both quantitative and qualitative aspects of hemostatic system components [11]. Available epidemiological data, although limited, shows that pediatric snakebite victims face twice the mortality risk and require double the frequency of antivenom administration compared to adult counterparts [12-14]. The increased severity in pediatric cases manifests through various pathophysiological mechanisms, primarily attributed to the elevated venom-to-body mass ratio, resulting in neurotoxicity, coagulopathy, and severe local tissue damage [15].

While snakebite envenomation is extensively studied in adults, research focusing on pediatric cases in rural South India remains limited. Given the unique physiological vulnerabilities of children and the high incidence of snakebites in our region, understanding the clinical profile and outcomes in this population is crucial. Therefore, this study was conducted to analyze the prevalence, clinical manifestations, and outcomes of snakebite envenomation among children in a rural tertiary care setting.

## Materials and methods

Study design and setting

This hospital-based retrospective observational study was conducted at Dhanalakshmi Srinivasan Medical College and Hospital, a tertiary care teaching hospital in the Perambalur district of Tamil Nadu, India. The institution serves as a major referral center for the surrounding rural communities of Perambalur and neighboring districts.

Data collection period

The study period extended from January 1, 2021, to December 31, 2023 (three years).

Sample size calculation

A retrospective study by Kshirsagar et al. analyzed 162 snakebite cases and reported diplopia as the predominant clinical manifestation, with a prevalence of 73.3% [16]. The minimum required sample size was calculated using the following formula: n = 3.84 × p × (1-p) / d^2^, where p = 0.733 (prevalence) and d = 0.07 (7% absolute precision). The calculation yielded a minimum sample size of 155 cases. Anticipating potentially incomplete medical records in our retrospective review, we incorporated a 25% adjustment, resulting in a final target sample size of 195 cases.

Inclusion criteria

For this study, we included all children below 16 years of age who were admitted to the Department of Pediatrics at Dhanalakshmi Srinivasan Medical College and Hospital with a diagnosis of snakebite during the study period. Only cases with complete medical records available in the hospital's Medical Records Department were considered for inclusion in the study.

Exclusion criteria

Cases were excluded if the patient was 16 years or older, if the medical records were incomplete or unavailable, if the snakebite was managed on an outpatient basis, or if the final diagnosis was revised to other causes during the course of treatment. Additionally, cases where patients were brought dead or left against medical advice before confirmation of snakebite diagnosis were not included in the analysis.

Data source

Data was extracted from the medical records maintained in the Medical Records Department of Dhanalakshmi Srinivasan Medical College and Hospital using a structured data collection form.

Data collection procedure

Demographic and epidemiological data were extracted from medical records, including patient age, gender, geographical location of residence, site of snakebite, and species identification when available. Specific bite-related information documented included the anatomical site of the bite and snake species (when identified). Time-critical parameters were carefully documented, including the interval between the snakebite and hospital presentation, any first aid measures received, and the time of onset of clinical manifestations. The clinical documentation encompassed comprehensive details of local symptoms (including fang marks and wound characteristics), systemic manifestations (hematotoxicity and neurotoxicity features), and vital parameters at presentation.

Treatment details were systematically collected, including the administration of polyvalent ASV as per WHO guidelines, dosing protocols, requirements of supportive therapies, and duration of hospital stay. The clinical course was tracked from admission through discharge or demise, with particular attention to the development of complications. To ensure data accuracy and completeness, two independent researchers extracted the information using a structured data abstraction form. Any discrepancies were resolved through consultation with a senior pediatrician.

Ethical consideration

The study protocol received approval from the Institutional Ethics Committee of Dhanalakshmi Srinivasan Medical College and Hospital (approval number: IECHS/IRCHS/No. 391A) with a waiver of individual informed consent because of its retrospective nature and the use of existing medical records. All patient data were de-identified during data extraction and analysis to maintain confidentiality, with each case assigned a unique study identifier. Data collection, storage, and analysis were conducted in accordance with institutional privacy protocols and the Declaration of Helsinki guidelines. Access to medical records was limited to authorized study personnel, and the collected information was used only for the stated research objectives. There was no inclusion of personally identifiable information in the analysis or manuscript.

Data analysis

Statistical analyses were performed using SPSS Statistical software version 23.0 (IBM Corp., Armonk, NY). Descriptive analyses included frequency distributions and percentages for categorical variables, while continuous variables were summarized using means and standard deviations. Comparative analyses between categorical variables were conducted using Pearson's chi-square test, with Fisher's exact test applied when expected cell frequencies were less than 5. A two-tailed p-value of <0.05 was considered statistically significant for all analyses.

## Results

In our study, 202 study participants were included in total. The age-stratified analysis revealed a predominance of snakebite cases in the 9-12 year age group (n = 110, 54.5%), followed by the 5-8 year age group (n = 62, 30.7%). Notably, children under four years comprised the smallest proportion of cases (n = 30, 14.8%). We observed a notable male predominance among snakebite victims, with males accounting for more than two-thirds of cases (n = 148, 73.3%), while females represented approximately one-third of the study population (n = 54, 26.7%). With regard to religious distribution, Hindus comprised the majority of cases (n = 152, 75.2%), followed by Christians (38, 18.9%). Table 1 shows the sociodemographic details of the study participants.

**Table 1:** Sociodemographic details of the study participants (N = 202)

Sociodemographic variables	Frequency	Percentage
Age of the participants		
≤4 years	30	14.8
5-8 years	62	30.7
9-12 years	110	54.5
Gender		
Male	148	73.3
Female	54	26.7
Religion		
Hindu	152	75.2
Muslim	12	5.9
Christian	38	18.9

Analysis of snake species involved in pediatric envenomation revealed that unidentified snakes constituted the largest proportion of cases (n = 72, 35.6%). Among identified species, vipers were the most frequent offending snake (n = 65, 32.2%), followed by cobra (n = 53, 26.2%). Krait bites were relatively uncommon, accounting for only 5.9% (n = 12) of cases. Notably, identified venomous snakes collectively represented 64.4% of all cases, highlighting the significant burden of venomous snakebites in our study population.

Analysis of bite site distribution revealed that lower limbs were predominantly affected, accounting for more than half of the cases (n = 108, 53.5%). Upper limb involvement was observed in approximately one-fourth of the cases (n = 48, 23.8%). Trunk and facial bites were less frequent, comprising 13.9% (n = 28) and 8.8% (n = 18) of cases, respectively.

The seasonal distribution analysis revealed a predominant clustering of cases from January to April, accounting for more than half (n = 106, 52.5%) of all pediatric snakebite presentations. The period between September and December recorded the second highest frequency with approximately one-third of cases (n = 66, 32.7%), while May to August showed the lowest incidence with only 14.8% (n = 30) of cases. Table 2 shows the characteristics of snakebites.

**Table 2:** Characteristics of snakebites

Variables	Frequency	Percentage
Snake species		
Cobra	53	26.2
Krait	12	5.9
Unidentified	72	35.6
Viper	65	32.2
Seasonal variation		
January to April	106	52.5
May to August	30	14.8
September to December	66	32.7
Site of bite		
Face	18	8.8
Lower limb	108	53.5
Trunk	28	13.9
Upper limb	48	23.8

Analysis of clinical manifestations revealed a diverse spectrum of symptoms and signs among the study population (Table 3). Hematological and renal manifestations were predominant, with hematuria being the most frequent presentation (n = 112, 55.4%), followed closely by oliguria (n = 102, 50.5%). Eighty (39.6%) children were diagnosed with renal failure. Local manifestations were also common, with pain and swelling affecting nearly half of the patients (n = 94, 46.5%) and oozing from the bite site observed in 29.7% (n = 60) of cases.

**Table 3:** Clinical manifestations of snakebite envenomation in pediatric patients (N = 202)

Clinical manifestations	Frequency	Percentage
Hematuria	112	55.4
Oliguria	102	50.5
Swelling	94	46.5
Renal failure	80	39.6
Oozing from the site	60	29.7
Epistaxis	52	25.7
Drooping of eyelid	38	18.8
Respiratory paralysis	26	12.9
Hematemesis	20	9.9
Melena	12	5.9

Neurotoxic manifestations were notable, with ptosis (drooping of the eyelid) present in 18.8% (n = 38) of cases and respiratory paralysis, a severe complication, affecting 12.9% (n = 26) of patients. Bleeding manifestations were observed in various forms: epistaxis (52.7%), hematemesis (20.9%), and melena (12.9%), indicating significant coagulopathy in these cases.

Analysis of the time interval between snakebite and ASV administration revealed that the majority of patients (n = 120, 59.4%) received treatment within the critical first six hours following the bite, which aligns with recommended emergency management protocols. Approximately one-third of cases (n = 60, 29.7%) received ASV between seven and 12 hours post-bite, while a smaller proportion (n = 26, 12.9%) had delayed presentation and treatment beyond 12 hours.

The total ASV requirement showed considerable variation among patients. Nearly half of the cases (n = 94, 46.6%) required 5-10 vials, representing the most common dosage range. Higher doses were necessary in the remaining cases, with 20.8% (n = 42) requiring 11-15 vials, 15.8% (n = 32) needing 16-20 vials, and 16.8% (n = 34) requiring more than 20 vials, showing severe envenomation requiring intensive intervention. Table 4 shows the anti-snake venom administration pattern in pediatric snakebite cases.

**Table 4:** ASV administration pattern in pediatric snakebite cases ASV: anti-snake venom

ASV administration pattern	Frequency	Percentage
Time between bite and ASV administration		
≤6 hours	120	59.4
7 to 12 hours	60	29.7
>12 hours	26	12.9
Total number of ASV vials given		
5 to 10 vials	94	46.6
11 to 15 vials	42	20.8
16 to 20 vials	32	15.8
>20 vials	34	16.8

**Figure 1:**
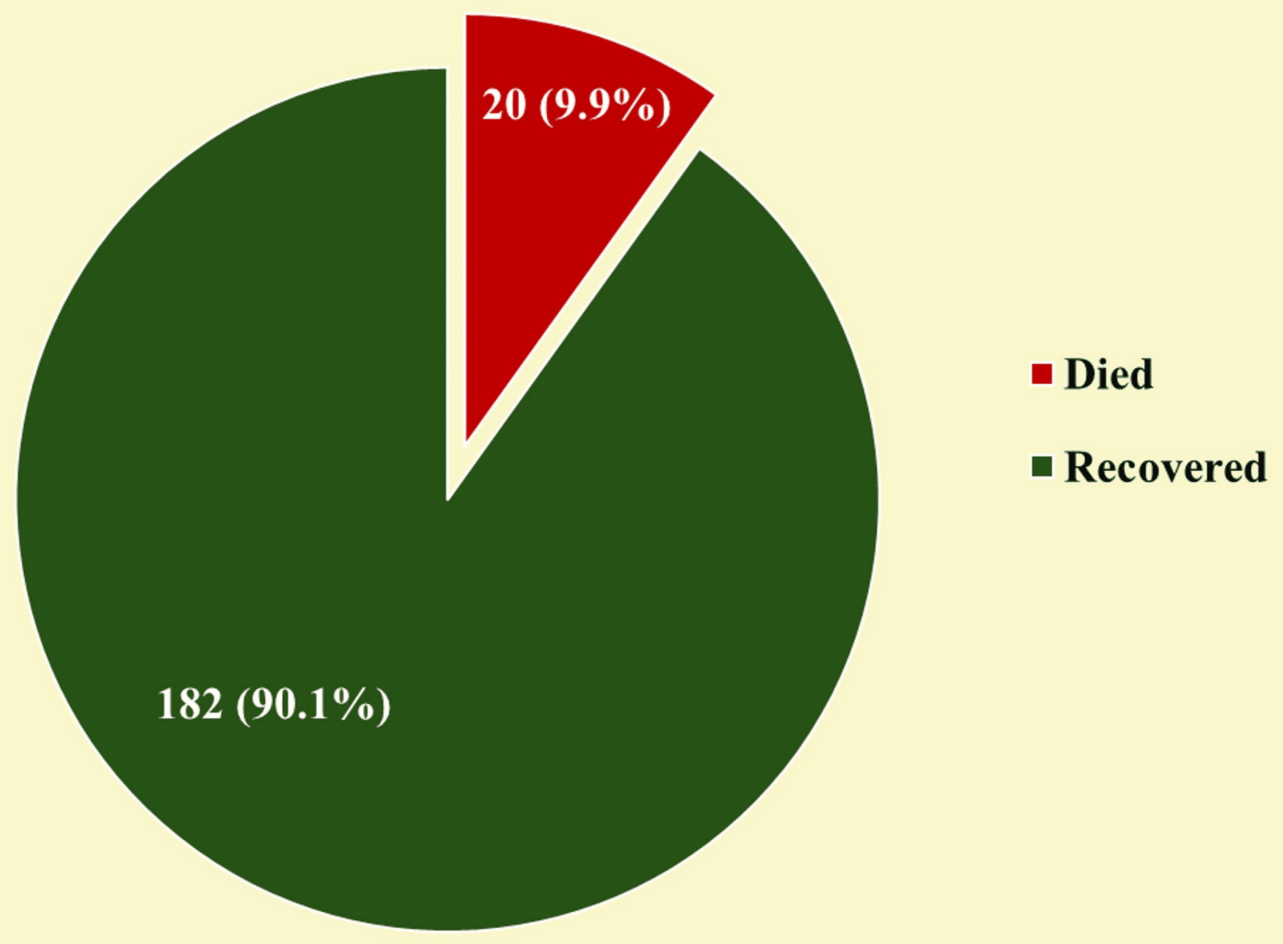
Clinical outcomes of pediatric snakebite cases (N = 202)

The pie chart illustrates the clinical outcomes of pediatric snakebite cases in our study population (Figure 1). The analysis reveals that the majority of patients (n = 182, 90.1%) recovered successfully following treatment. However, there was a mortality rate of 9.9% (n = 20) despite medical intervention. It underscores the significant potential severity of snakebite envenomation in pediatric populations.

Statistical analysis of factors associated with mortality in pediatric snakebite cases revealed several significant associations, evaluated using the chi-square test or Fisher's exact test as appropriate (Table 5). Demographic factors such as age distribution (p = 0.786) and gender (p = 0.378) showed no statistically significant association with mortality.

**Table 5:** Association between clinical variables and mortality outcomes in pediatric snakebite cases: univariate analysis (N = 202) *Statistically significant ASV: anti-snake venom

Variables		Died (20)	Recovered (182)	Test value	p-value
Age group	≤4 years	2 (6.7)	28 (93.3)	0.480	0.786
	5-8 years	6 (9.7)	56 (90.3)		
	9-12 years	12 (10.9)	98 (89.1)		
Gender	Female	7 (13)	47 (87)	0.774*	0.378
	Male	13 (8.8)	135 (91.2)		
Snake species	Cobra	1 (1.9)	52 (98.1)	15.530	0.0014
	Krait	1 (8.3)	11 (91.7)		
	Unidentified	15 (20.8)	57 (79.2)		
	Viper	3 (4.6)	62 (95.4)		
Site of bite	Face	1 (5.6)	17 (94.4)	8.184	0.042
	Lower limb	15 (13.9)	93 (86.1)		
	Trunk	4 (14.3)	24 (85.7)		
	Upper limb	0 (0)	48 (100)		
Renal failure	Yes	18 (22.5)	62 (77.5)	23.569	0.001
	No	2 (1.6)	120 (98.4)		
Respiratory paralysis	Yes	10 (38.5)	16 (61.5)	27.286*	0.001
	No	10 (5.7)	166 (94.3)		
Oliguria	Yes	1 (0.9)	101 (99.1)	18.379	0.001
	No	19 (19)	81 (81)		
Drooping of eyelid	Yes	17 (44.7)	21 (55.3)	63.671	0.001
	No	3 (1.8)	161 (98.2)		
Swelling	Yes	9 (9.6)	85 (90.4)	0.021*	0.884
	No	11 (10.2)	97 (89.8)		
Epistaxis	Yes	6 (11.5)	46 (88.5)	0.210*	0.646
	No	14 (9.3)	136 (90.7)		
Time between bite and ASV administration	≤6 hours	1 (0.8)	119 (99.2)	37.224	0.001
	7 to 12 hours	9 (16.1)	47 (83.9)		
	>12 hours	10 (38.5)	16 (61.5)		

Snake species identification showed a significant correlation with mortality (p = 0.0014), with unidentified snakebites having the highest mortality rate (n = 15, 20.8%), followed by krait (n = 1, 8.3%), viper (n = 3, 4.6%), and cobra (n = 1, 1.9%). The anatomical site of bites significantly influenced outcomes (p = 0.042), where lower limb bites (n = 15, 13.9%) and trunk bites (n = 4, 14.3%) showed higher mortality rates, while upper limb bites had no mortality.

Among clinical complications, several factors showed strong associations with mortality. Renal failure was significantly associated with adverse outcomes (p = 0.001), with a 22.5% mortality rate among affected patients. Respiratory paralysis showed a marked impact on survival (p = 0.001), with a 38.5% mortality rate in affected cases. Notably, the presence of drooping of eyelids, a neurotoxic manifestation, was significantly associated with higher mortality (44.7%, p = 0.001). Interestingly, oliguria showed an inverse relationship (p = 0.001), with lower mortality (0.9%) in patients presenting with this symptom.

The time between snakebite and ASV administration emerged as a crucial determinant of outcome (p = 0.001). Early treatment within six hours showed the lowest mortality rate (0.8%), while intermediate delays (7-12 hours) resulted in 16.1% mortality, and late treatment (beyond 12 hours) was associated with the highest mortality rate of 38.5%. In contrast, local manifestations such as swelling (p = 0.884) and bleeding manifestations such as epistaxis (p = 0.646) did not show a significant association with mortality.

Table 6 shows that the analysis of factors associated with major complications revealed significant correlations with snake species, bite site, and time to ASV administration. Neurotoxic complications (respiratory paralysis and ptosis) were significantly more common in cobra (28.3% and 37.7%, respectively) and krait bites (41.7% and 50%, respectively) compared to viper bites (p < 0.001). Renal failure showed highest prevalence in unidentified snakebites (52.8%, p = 0.003). Delayed ASV administration beyond 12 hours was associated with significantly higher rates of all complications (p < 0.001): renal failure (68.2%), respiratory paralysis (36.4%), and ptosis (50%). Demographic factors including age and gender showed no significant association with complication rates.

**Table 6:** Association between clinical variables and major complications in pediatric snakebite cases (N = 202) *Statistically significant

Variables	Renal failure	Respiratory paralysis	Drooping of eyelid
Age group			
≤4 years (n = 30)	10 (33.3%)	2 (6.7%)	4 (13.3%)
5-8 years (n = 62)	25 (40.3%)	8 (12.9%)	12 (19.4%)
9-12 years (n = 110)	45 (40.9%)	16 (14.5%)	22 (20%)
p-value	0.742	0.521	0.698
Gender			
Female (n = 54)	20 (37%)	8 (14.8%)	12 (22.2%)
Male (n = 148)	60 (40.5%)	18 (12.2%)	26 (17.6%)
p-value	0.654	0.621	0.459
Snake species			
Cobra (n = 53)	12 (22.6%)	15 (28.3%)	20 (37.7%)
Krait (n = 12)	4 (33.3%)	5 (41.7%)	6 (50%)
Unidentified (n = 72)	38 (52.8%)	4 (5.6%)	8 (11.1%)
Viper (n = 65)	26 (40%)	2 (3.1%)	4 (6.2%)
p-value	0.003*	<0.001*	<0.001*
Site of bite			
Face (n = 18)	5 (27.8%)	4 (22.2%)	6 (33.3%)
Lower limb (n = 108)	48 (44.4%)	12 (11.1%)	18 (16.7%)
Trunk (n = 28)	12 (42.9%)	4 (14.3%)	6 (21.4%)
Upper limb (n = 48)	15 (31.3%)	6 (12.5%)	8 (16.7%)
p-value	0.032*	0.048*	0.041*
Time to ASV administration			
≤6 hours (n = 120)	35 (29.2%)	8 (6.7%)	12 (10%)
7-12 hours (n = 60)	30 (50%)	10 (16.7%)	15 (25%)
>12 hours (n = 22)	15 (68.2%)	8 (36.4%)	11 (50%)
p-value	<0.001*	<0.001*	<0.001*

## Discussion

Our study of 202 pediatric snakebite cases revealed a predominance in children aged 9-12 years (54.5%) with male gender predominance (73.3%). Unidentified snakes caused the most envenomation (35.6%), followed by vipers (32.2%) and cobras (26.2%). Lower limb bites were the most frequent (53.5%), with peak occurrence during January-April (52.5%). The most common clinical manifestations were hematuria (55.4%), oliguria (50.5%), and renal failure (39.6%). Neurotoxic manifestations included ptosis (18.8%) and respiratory paralysis (12.9%). Most patients (59.4%) received antivenom within six hours, with 46.6% requiring 5-10 vials. The overall mortality rate was 9.9%. Mortality was significantly associated with snake species identification, anatomical bite site, renal failure, respiratory paralysis, and ptosis. Time to antivenom administration was crucial, with mortality increasing from 0.8% for treatment within six hours to 38.5% for delays beyond 12 hours.

The study demonstrates a clear male predominance (73.3%) among snakebite victims, a finding consistently observed across multiple studies. Similar male preponderance was reported by Kshirsagar et al. (60.49%) [16] and Suganthi et al. (64.8%) [17]. This gender disparity likely reflects higher outdoor activity levels and risk-taking behavior among male children in the studied age groups. The peak age group of 9-12 years (54.5%) in our study corresponds closely with the study findings of Karunanayake et al., where children aged 6-12 years constituted 48% of cases, suggesting this age group's increased vulnerability because of outdoor activities and possibly reduced adult supervision [18].

The challenge of snake species identification emerged as a significant concern in our study, with unidentified snakes accounting for 35.6% of cases. While this percentage is considerable, it is notably lower than the findings of Shrestha, where unidentified snakes accounted for 61% of cases [14]. This persistent difficulty in snake identification across different studies highlights a critical gap in snakebite management. Among identified species, our study found vipers (32.2%) as the second most common cause, followed by cobras (26.2%). This pattern aligns with the findings of Suganthi et al., where viper bites were predominant among identified species [17]. The variation in snake species distribution across studies likely reflects regional differences in snake populations and ecology, emphasizing the need for region-specific management protocols.

The anatomical distribution of snakebites in our study showed a clear predominance of lower limb involvement (53.5%). This finding is consistently reported across multiple studies, although with varying frequencies: Kshirsagar et al. (74.04%) [16], Suganthi et al. (85%) [17], and Paudel et al. (32%) [19]. This consistency across studies suggests that preventive measures should particularly focus on protecting the lower extremities, especially during high-risk activities and seasons. Our study's observation of peak incidence during January-April (52.5%) provides valuable information for healthcare resource allocation and community awareness programs.

The spectrum of clinical manifestations in our study revealed interesting patterns that merit careful consideration. We observed high rates of hematological and renal manifestations, with hematuria (55.4%), oliguria (50.5%), and renal failure (39.6%) being predominant. This differs from the findings of Kshirsagar et al., where neurological manifestations such as diplopia (73.3%) and respiratory distress (66.7%) were more frequent [16]. Our study found relatively lower rates of neurotoxic manifestations, with ptosis at 18.8% and respiratory paralysis at 12.9%. This contrasts with the study of Shrestha, where respiratory paralysis affected 85% of cases [14]. These variations might be attributed to differences in prevalent snake species, their venom properties, and patterns of clinical presentation across regions.

A crucial finding of our study is the strong correlation between treatment timing and mortality. The dramatic increase in mortality from 0.8% with treatment within six hours to 38.5% with delays beyond 12 hours emphatically shows the critical importance of early intervention. Most patients in our study (59.4%) received antivenom within six hours, with 46.6% requiring 5-10 vials, highlighting the substantial variation in antivenom requirements. This finding underscores the need for adequate antivenom availability and standardized dosing protocols in pediatric cases.

Our overall mortality rate of 9.9% falls within the range reported by other studies: Karunanayake et al. (11%) [18], Suganthi et al. (11.1%) [17], and Paudel et al. (16%) [19], although significantly lower than Shrestha's report of 28% [14]. The variation in mortality rates across studies might reflect differences in healthcare accessibility, timing of intervention, and prevalent snake species. Our study identified several factors significantly associated with mortality, including snake species identification, anatomical bite site, renal failure, respiratory paralysis, and ptosis. The risk factors for mortality identified in our study align with several previous reports. Shrestha's study similarly identified unidentified snake species and specific anatomical bite locations as mortality risk factors [14]. Our finding of significant associations between mortality and renal failure, respiratory paralysis, and ptosis provides valuable prognostic indicators for clinical management. This corresponds with the observations of Paudel et al., where similar complications were associated with poor outcomes [19].

The statistical significance of various clinical factors associated with mortality in our study provides important insights for risk stratification and management. The higher mortality among cases with unidentified snake species (20.8%) compared to identified species highlights the critical importance of accurate snake identification in guiding appropriate treatment. The significant association between the site of anatomical bite and mortality, with higher rates in lower limb and trunk bites, suggests the need for vigilance in such cases.

Clinical implications

Our findings have several important implications for clinical practice. First, the strong correlation between delayed antivenom administration and increased mortality emphasizes the critical importance of establishing efficient emergency response systems and referral networks. Healthcare facilities must maintain adequate antivenom supplies and develop clear protocols for its rapid administration in pediatric cases. Second, the high incidence of renal complications suggests the need for early and regular monitoring of renal function in all pediatric snakebite cases, with particular attention to cases showing early signs of renal involvement. Third, the significant proportion of unidentified snake species necessitates the development of standardized management protocols for cases where the snake species cannot be identified. These protocols should consider regional snake species distribution patterns and common envenomation syndromes.

The observed patterns of clinical manifestations and complications indicate the need for a comprehensive monitoring approach. Regular assessment of both local and systemic manifestations, particularly focusing on renal and neurological parameters, is crucial for early detection and management of complications. The relatively high incidence of neurotoxic manifestations, even in non-neurotoxic snakebites, suggests the need for vigilant neurological monitoring in all cases.

The relationship between bite sites and outcomes highlights the importance of site-specific management protocols. Lower limb bites, being most common and associated with higher mortality in our study, require particular attention to compartment syndrome and local complications while maintaining vigilance for systemic manifestations. The seasonal pattern of snakebites suggests the need for increased resource allocation and preparedness during peak seasons.

Furthermore, our findings emphasize the importance of a structured approach to pediatric snakebite management, including early recognition of complications, appropriate ASV dosing, and careful monitoring of treatment response. The observation that most cases required 5-10 vials of ASV provides valuable guidance for initial dosing strategies, although this should be adjusted based on individual clinical response and local protocols.

The mortality patterns observed in our study, particularly the dramatic difference based on time to ASV administration, underscore the critical importance of public education about early hospital presentation and the need for robust emergency transport systems. These findings suggest that community-level interventions focusing on early recognition and rapid transport of snakebite victims could significantly improve outcomes.

Strength of the study

This study demonstrates significant clinical relevance through its comprehensive analysis of pediatric snakebite cases in a rural tertiary care setting. The research provides valuable insights into age-stratified patterns and envenomation characteristics, essential for healthcare planning and resource allocation. The detailed documentation of clinical manifestations and their correlation with outcomes offers crucial guidance for the early recognition and management of severe cases. The study's analysis of anti-snake venom timing and dosage patterns provides practical treatment protocols, while the identification of specific prognostic factors enables better risk stratification and patient monitoring. These findings are particularly valuable for improving emergency care in resource-limited settings.

Limitations

Being a single-center, hospital-based study, it potentially excludes cases managed at primary centers, those seeking traditional medicine, and fatal cases before hospital arrival, introducing selection bias. The retrospective nature led to documentation challenges, particularly in snake species identification (35.6% unidentified) and standardization of clinical assessment intervals. The absence of long-term follow-up data limited our understanding of delayed complications and quality of life outcomes. Additional limitations include incomplete documentation of pre-hospital interventions, underlying health conditions, and traditional treatments received. The analysis did not fully consider time-dependent variables, such as seasonal changes in healthcare accessibility and the impact of socioeconomic factors on treatment delays. These limitations should be addressed in future prospective studies.

## Conclusions

This study examined the clinical characteristics and outcomes of snakebite cases among children, with a small proportion of cases resulting in mortality. The clinical profile demonstrated varied presentations, with hematuria, oliguria, and local pain and swelling being the predominant manifestations. Demographic patterns showed no significant influence on mortality outcomes. However, the envenomation characteristics, particularly unidentified snake species, were associated with higher mortality rates. The spectrum of complications included both neurotoxic manifestations (ptosis and respiratory paralysis) and systemic complications (renal failure), which significantly influenced outcomes. Analysis of risk factors identified delayed ASV administration beyond six hours as a critical determinant of mortality. The study established that time to treatment, the presence of neurotoxic features, and the development of complications were key prognostic factors in pediatric snakebite cases, while local manifestations and demographic factors had a minimal impact on mortality.

## References

[1] Essafti M, Fajri M, Rahmani C, Abdelaziz S, Mouaffak Y, Younous S (2022). Snakebite envenomation in children: an ongoing burden in Morocco. Ann Med Surg (Lond).

[2] Kasturiratne A, Wickremasinghe AR, de Silva N (2008). The global burden of snakebite: a literature analysis and modelling based on regional estimates of envenoming and deaths. PLoS Med.

[3] Minghui R, Malecela MN, Cooke E, Abela-Ridder B (2019). WHO's snakebite envenoming strategy for prevention and control. Lancet Glob Health.

[4] Seifert SA, Armitage JO, Sanchez EE (2022). Snake envenomation. N Engl J Med.

[5] Zdenek CN, Rodrigues CF, Bourke LA, Tanaka-Azevedo AM, Monagle P, Fry BG (2023). Children and snakebite: snake venom effects on adult and paediatric plasma. Toxins (Basel).

[6] Sood A, Rana A, Kumar P (2020). Epidemiological and clinical profile of paediatric snake bite patients at a tertiary care centre of Himachal Pradesh, India. Int J Contemp Pediatr.

[7] (2024). Guidelines for the management of snakebites, 2nd ed. https://iris.who.int/handle/10665/249547.

[8] Newman WJ, Moran NF, Theakston RD, Warrell DA, Wilkinson D (1997). Traditional treatments for snake bite in a rural African community. Ann Trop Med Parasitol.

[9] Vinchu SS, Ezhilarasan VK (2017). An epidemiological study of clinical outcome of snakebite in children. Int J Med Paediatr Oncol.

[10] Parrish HM, Goldner JC, Silberg SL (1965). Comparison between snakebites in children and adults. Pediatrics.

[11] Monagle P, Barnes C, Ignjatovic V (2006). Developmental haemostasis. Impact for clinical haemostasis laboratories. Thromb Haemost.

[12] Wood D, Sartorius B, Hift R (2016). Snakebite in north-eastern South Africa: clinical characteristics and risks for severity. S Afr Fam Pract.

[13] Sankar J, Nabeel R, Sankar MJ, Priyambada L, Mahadevan S (2013). Factors affecting outcome in children with snake envenomation: a prospective observational study. Arch Dis Child.

[14] Shrestha B (2011). Outcomes of snakebite envenomation in children. J Nepal Paedtr Soc.

[15] Le Geyt J, Pach S, Gutiérrez JM (2021). Paediatric snakebite envenoming: recognition and management of cases. Arch Dis Child.

[16] Kshirsagar VY, Ahmed M, Colaco SM (2013). Clinical profile of snake bite in children in rural India. Iran J Pediatr.

[17] Suganthi V, Santhi K, Nandhini S (2018). Clinico-epidemiological profile of snake bite in children in a tertiary care hospital, South India. Pediatr Rev Int J Pediatr Res.

[18] Karunanayake RK, Dissanayake DM, Karunanayake AL (2014). A study of snake bite among children presenting to a paediatric ward in the main Teaching Hospital of North Central province of Sri Lanka. BMC Res Notes.

[19] Paudel KM, Poudyal VP, Rayamajhi RB, Budhathoki SS (2015). Clinico-epidemiological profile and outcome of poisonous snake bites in children using the WHO treatment protocol in Western Nepal. J Nobel Med Coll.

